# Tracking Restoration of Park and Urban Street Settings in Coronary Artery Disease Patients

**DOI:** 10.3390/ijerph13060550

**Published:** 2016-05-31

**Authors:** Regina Grazuleviciene, Jone Vencloviene, Raimondas Kubilius, Vytautas Grizas, Asta Danileviciute, Audrius Dedele, Sandra Andrusaityte, Astra Vitkauskiene, Rasa Steponaviciute, Mark J. Nieuwenhuijsen

**Affiliations:** 1 Department of Environmental Science, Vytauto Didziojo Universitetas, K. Donelaicio 58, Kaunas 44248, Lithuania; j.vencloviene@gmf.vdu.lt (J.V.); a.danileviciute@gmf.vdu.lt (A.D.); a.dedele@gmf.vdu.lt (A.D.); s.andrusaityte@gmf.vdu.lt (S.A.); 2 Department of Cardiology, Medical Academy, Lietuvos Sveikatos Mokslu Universitetas, Eivenių 2, Kaunas 50161, Lithuania; raimondas@efarmacija.lt; 3 Institute of Cardiology, Medical Academy, Lietuvos Sveikatos Mokslu Universitetas, Kaunas 50161 Lithuania; vytautasgri@gmail.com; 4 Department of Laboratory Medicine, Medical Academy, Lietuvos Sveikatos Mokslu Universitetas, Eiveniu Str. 2, Kaunas 50028, Lithuania; astra.vitkauskiene@kaunoklinikos.lt (A.V.); rasastepkons@gmail.com (R.S.); 5 Centre for Research in Environmental Epidemiology (CREAL), Doctor Aiguader 88, Barcelona 08003, Spain; mnieuwenhuijsen@creal.cat

**Keywords:** physical activity, green space, urban environment, cardiovascular disease, cortisol, hemodynamic parameters

## Abstract

The physiological effects of natural and urban environments on the cardiovascular system of coronary artery disease (CAD) patients are not fully understood. This controlled field study examines the effects of restorative walking in a park *vs.* in an urban street environment on CAD patients’ stress parameters and cardiac function. *Methods:* Twenty stable CAD patients were randomly allocated to 7 days controlled walking in a city park or in an urban street environment group. The relationship between different environmental exposures and health effects was analyzed using Wilcoxon signed-rank test and exact Mann-Whitney U test. *Results:* The mean reduction in cortisol levels and negative effects after the walk on the first day was greater in the city park than in the urban street exposed group, while a reduction in negative effects in the urban group were greater after seven days. The reduction in diastolic blood pressure (DBP) in the park group was evident on the seventh day before the walk (−4 mm Hg, *p* = 0.031) and 60 min after the walk (−6.00 mm Hg, *p* = 0.002). The cortisol slope was negatively associated with the DBP changes (*r* = −0.514, *p* < 0.05). *Conclusions:* Physical activity in a green environment with noise and air pollution levels lower than in an urban environment has a greater positive effect on CAD patients’ stress level and hemodynamic parameters. Mitigating green environmental influences may allow urban residents to maintain health and reduce disability.

## 1. Introduction

Environmental non-communicable diseases, such as coronary artery disease (CAD), represent a growing global public health emergency. The environment might influence disease course through exposures to physical, chemical, social, and psychosocial risk factors, and through related changes in behavior in response to those factors. Assessment of the potential contribution of environmental factors to CAD course is important since many such factors are modifiable either through individual behaviors or government regulation, and early intervention has the potential for significant public health benefit.

This study is based on some evidence that physical activity might contribute to the prevention of CAD, improve prognosis of the patients, and decrease depressive symptoms [1,2]. However, relatively little attention has been paid to the specific environmental characteristics, such as the laboratory, type of urban environment (park or street) and the ambient air quality of the space where physical activity took place during the restorative treatment. While there is some evidence that exposure to a natural environment may improve mood and reduce stress, the effect of green space exposure on physiological responses has been proven to be inconsistent [3,4]. Studies into the effects of exercise-based cardiac rehabilitation on hemodynamic parameters in post-myocardial infarction (post-MI) patients have also revealed heterogeneous results [5,6]. CAD is associated with an increased stress and myocardial overload due to the elevated heart rate (HR), and it is contractility induced by sympathetic hyperactivity. Stress is associated with an increased negative effect [3] and through the activation of the sympathetic nervous system and the hypothalamic-pituitary-adrenal axis can influence cardiac function [7].

So far, the results of studies into the impact of the natural environment on stress markers through cortisol have been inconsistent, and the restorative effects of the natural environment on physiological and psychological parameters may differ depending on their characteristics [7,8]. The findings suggest that even short-term visits to nature areas, such as urban parks, and urban woodland, have a positive effect on perceived stress relief compared to the built-up environment, but there were no differences in the decrease of salivary cortisol levels during the experiment [9]. A link between the use of environmental self-regulation strategies and restorative outcomes has also been reported [10].

Some epidemiologic studies have provided evidence showing associations between increases in exposure to ambient PM_2.5_ and higher noise levels and increases in blood pressure in adults, especially within communities with elevated levels of exposure [11,12]. Possible pathways through which PM could elevate blood pressure and promote CAD events include the increase of pollution-induced systemic oxidative stress/inflammation, altered autonomic nervous system balance, and systemic pro-inflammatory responses, causing arterial remodeling [13]. An association between exposure to air pollution and the progression of atherosclerosis point to PM-related blood pressure increases as one possible mechanism by which air pollution may contribute to the acceleration of cardiovascular disease (CVD) development [12,14,15]. Therefore, the physiological responses to a park environment compared to an urban environment can, due to lower air pollution levels, have greater positive effects on cardiovascular responses.

The present study aimed to investigate whether walking in a park has a greater positive effect on CAD patients’ stress parameters and cardiac function than walking in an urban environment. This study used objective measures of the environment parameters where the physical activity was conducted, such as the levels of the main ambient air pollutants and noise. Moreover, the physiological response to controlled physical activity in the different environments was also measured as salivary cortisol levels, and cardiac function parameters. This study inclusion criteria, all exclusions, measures, experimental conditions, and sample size determination (data collection stopped once a predetermined sample size was reached) have been reported [16]. The study was conducted as part of the European Commission 7th Framework Programme Positive Health Effects of the Natural Outdoor Environment in Typical Populations in Different Regions in Europe (EC FP7 PHENOTYPE) project [17]. This controlled field study is the first to investigate whether the restorative effects of the natural environment on cardiac function of CAD patients’ is associated with a reduction in stress level and improvement of mood.

## 2. Materials and Methods

This study was conducted in Kaunas city, Lithuania. The study included 20 male and female Kaunas city residents with CAD. Written informed consent was obtained from each participant. The study was conducted in accordance with the Declaration of Helsinki, and the protocol was approved by the Ethics Committee of Lithuania 2012-04-30 No. 6B-12-147 (Project identification code FP7-282996). The participants’ characteristics, patients’ randomization and investigation scheme of the urban street and the park environment exposure groups were presented in a previous publication [16].

The age of the participants of both sexes ranged between 45 and 75 years (mean age = 62.3 ± 12.6 years). The urban exposure (30 min, *n* = 10) was a busy street behind the Clinic of Cardiology (10,000 cars/day). The green exposure (*n* = 10) was a beautiful pine park terrain cure of the Neris river coast (about 70% of their land covered with pine) with pathways for various gradients of ascent, located within a 5-min walk of the Clinic of Cardiology. The pine park is accessed through the Clinic park with lawn squares (in total, the route was 30 min of green exposure). Both the urban street and park route was a round trip, single walk; however walking speed was controlled by a trained nurse to reach the personal capacity load determined during treadmill testing.

**Figure 1:**
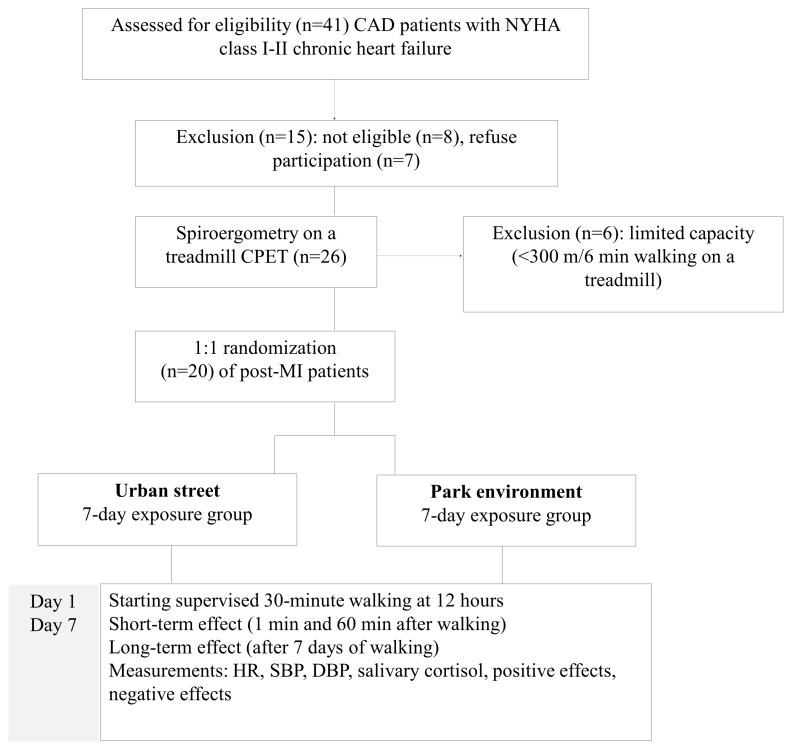
Randomization and investigation scheme of the urban street and the park environment exposure groups.

In this study, together with the hemodynamic parameters, we evaluated the walking effects on stress levels (by salivary cortisol concentration) and mood (positive effects (PE) and negative effects (NE). The patient groups were similar—both clinically and in terms of their residential environmental characteristics and physical activity. Data collection took place at the Clinic between 12:00 and 15:00 during the vegetation period May–September 2013. As detailed in Figure 1, the participants were first screened for eligibility using the post-MI patient register database.

Eligible participants were invited to come to the clinic at 12:00 for their first measurements, and to refrain from consuming caffeine or food for at least 60 min prior to their arrival. Exercise capacity testing using a treadmill and electrocardiogram (ECG) monitoring was performed at baseline. Walking intensity in the natural environment was estimated to be 10% lower than the capacity determined during treadmill testing. For the next 7 consecutive days following baseline (before walking) measurements and clinical investigation, the participants were directed to the natural environment and completed a 30-min walk along a pre-designated route with measurements repeated at 1 min and 60 min after the initial exposure.

The conditions of the experiment included the first hemodynamic measurements using a treadmill, urban-street exposure for 30 min on 7 consecutive days or green exposure in a pine forest for 30 min on 7 consecutive days, and the second hemodynamic measurements using a treadmill. We studied the short-term (1 min and 60 min after the walk) and cumulative 7-day effects of walking on the following hemodynamic parameters: heart rate (HR), systolic blood pressure (SBP), and diastolic blood pressure (DBP). To provide a physiological measure of stress, saliva samples for cortisol levels were collected 3 times per day: before walking, immediately following the 30-min exposure (1 min after the walk) and 60 min after the end of the exposure. Salivary cortisol analysis was carried out using enzyme immunoassay for the *in vitro* diagnostic quantitative determination of cortisol levels (μg/dL) in a 37-piece human saliva sample kit (IBL International GmbH, Hamburg, Germany) according to the protocol.

For the evaluation of mood scores, we used the Positive and Negative Effect Schedule (PANAS) [18]. Feelings and emotional state were assessed 3 times per day at the same time when cortisol and hemodynamic measurements were made. The measurements were performed on the 1st and the 7th day of the patients’ exposure to green or urban environments.

### Statistical Analysis

The statistical analysis involves before and after exposure comparisons in the same study subject and between differently exposed subjects. Quantitative variables were reported as median values and standard error. Bivariate relationships between the variables were explored using Spearman’s correlations. We used exact Fisher’s tests to compare the qualitative characteristics between patients exposed to the urban environment and to the park area. The normal distribution of variables and its logarithmic transformations were tested by using the Shapiro-Wilk test, and the unpaired and paired *t*-test was used to compare the means. The Mann-Whitney test was used for comparisons between differently exposed subjects and the Wilcoxon test was used for within-subject comparisons.

Statistical analyses were carried out using SPSS version 18.0 (SPSS Inc. Released 2009. PASW Statistics for Windows, Version 18.0. SPSS Inc., Chicago, IL, USA). *p*-values < 0.05 were considered statistically significant.

## 3. Results

### 3.1. Relationships between Cortisol, Mood, and Hemodynamic Parameters

The two experimental groups differed neither in their baseline characteristics, and cardiac function nor in their baseline salivary cortisol levels, PE, or NE scores (Table 1). However, there were significant differences in the characteristics between the two environments where physical activity took place during the experiment, with higher levels of air pollution (NO_2_ concentration by 3.84 μg/m^3^ higher, and PM_2.5_ concentration—by 6.41 μg/m^3^ higher) and noise (by 19.03 dBA higher) in the urban environment, compared with the park environment. There were non-significant differences in the air temperature during physical activity of both exposure groups; however, the relative humidity during the park exposure group patients’ walks was statistically significantly higher.

Bivariate correlations revealed that on day 1, higher mean cortisol concentrations in the total sample were positively associated with NE (*r* = 0.471, *p* < 0.05), and negatively associated–with SBP (*r* = −0.363, *p* < 0.1) (Table 2). The cortisol slope was negatively associated with HR decrease and DBP changes (*r* = −0.514, *p* < 0.05). There was a positive association between SBP on day 1 and PE (*r* = 0.483, *p* < 0.05), and a negative association between SBP changes and PE (*r* = −0.402, *p* < 0.05), whereas HR changes were positively associated with NE (*r* = 0.385, *p* < 0.1). In this study, stress measured as cortisol levels was not significantly related to PE scores; however, NE was positively related to cortisol levels (*r* = 0.471, *p* < 0.05). This indicates that NE states in CAD patients were stronger predictors of the stress level than PE was.

### 3.2. The First-Day and the Seven-Day Exposure Effects

On the first day, we observed a significant short-term effect of a 30-min walk in both the park and urban environment groups: there was an increase in SBP, DBP, and HR 1 min after the walk and the recovery of hemodynamic parameters after a 60-min rest. The median cortisol concentration and PE scores 60 min after the walk in an urban street tended to decrease and differed non-significantly from the baseline. However, in subjects exposed to the park environment, a statistically significant decrease in cortisol concentration, PE, and NE was found 1 min after the walk, and these changes were still evident 60 min after the walk (cortisol −2.89, *p* = 0.037; PE −2.00, *p* = 0.012; NE −1.00, *p* = 0.031) (Table 3). Such post-walk change in PE after a 30-min walk on the 1st day suggests that physical intensity (estimated to be by 10% below the capacity determined during treadmill testing) caused fatigue in post-MI patients.

We found a non-significant difference in resting hemodynamic parameters measured at baseline and 60 min after walking on days 1 and 7 in patients exposed to the urban environment (Table 4). In patients exposed to the park environment, there was evidence of a positive effect of training on hemodynamic parameters on day 7—*i.e.*, those walking in green environments showed a slight decrease in baseline SBP and a statistically significant decrease in baseline DBP by 4 mm Hg (*p* = 0.031). Moreover, 60 min after walking in the park, SBP on day 7 was reduced by 3 mm Hg (*p* = 0.131), and DBP was reduced by 6 mm Hg (*p* = 0.002), compared to the respective findings on day 1. After the 7-day exposure to natural and urban environments, in both groups there were non-significant changes in cortisol and PE measurements taken 60 min after walking, compared to those taken at baseline. In patients exposed to the urban street, changes in negative effects were statistically significant both at baseline and 60 min after walking.

## 4. Discussion

In this controlled field study, we investigated the restorative effects of short-term visits to the urban park and the urban street environment on post-MI patients’ stress and hemodynamic parameters. This study used objective measures of physiological response to physical activity (salivary cortisol levels, SBP, DBP, and HR) and subjective psychological measures (PE and NE). Moreover, in the environment where the physical activity was conducted, we measured the levels of the main ambient air pollutants, noise and meteorological conditions. This allowed us to study for the first time the physiological and psychological effects of walking in different natural environments on post-MI patients and to suggest a pathway by which natural environment might effect the impaired physiological parameters of CVD patients.

The findings of this study suggest that even 30 min visits to green areas have positive effects on stress relief and cardiac response compared to urban street environment. In patients exposed to the urban street, changes in negative effects were evident after 7-days of walking. These findings were partially influenced by the difference in ambient air pollution and noise levels between the two environments where physical activity took place during the experiment. Also, higher relative humidity during the walks of the park exposure group patients can have an impact on negative effects scores reduction after 7 days of walking. Our previously published data [16] and studies conducted by other authors [11,12] indicate that higher concentrations of ambient air pollutants and higher noise levels may have an impact on the risk of hypertension and that this, through an increase in SBP and DBP, may promote atherosclerosis and CAD.

The underlying mechanisms of the effect of the natural environment on the cardiovascular system are not fully understood. However, there is evidence that particle pollution during physical activity may promote arterial vasoconstriction through the imbalance of the cardiovascular autonomic nervous system [13,19], as well as an increase in peripheral blood pressure and heart rate [20]. Therefore, appropriate training in green environment might decrease blood pressure and—through the involvement of the sympathetic nervous system and the renin-angiotensin system—have a favorable effect on concomitant cardiovascular risk factors [21]. Our findings are in accordance with the results of studies that show that the natural settings differ in terms of their restorative quality [10,22], and urban green areas have greater perceived stress-reducing effects compared to the built-up environment [9]. The experiment revealed that the cortisol levels in post-MI patients were associated with an increase in NE, leading to a positive correlation between NE and cortisol. These findings are in line with the previous studies reporting positive relationships between cortisol and specific negative emotions in response to chronic stress [23], an attenuated negative emotional arousal in response to acute stress [3], and a non-significant relationship between cortisol and negative effect [24]. It seems that cortisol is more associated with the negative effects than with the positive ones, indicating that NE states were stronger predictors of the stress level than PE in CAD patients. The cortisol 60 min after the walk was statistically significantly decreased in the park exposure group. However, a study of healthy, non-smoking adults conducted in Helsinki, did not reveal any significant differences in the decrease in salivary cortisol levels after short-term visits to urban parks, urban woodland, and city centers [9].

Clinical trial data suggest that HR is influenced not only by the duration and intensity of training, but also by beta-blockers and other heart-rate lowering drugs used after acute myocardial infarction in patients with chronic heart failure [25]. The findings of the previous studies showed that 12-week walking produced a training effect on the cardiovascular system in people with mild hypertension [2], and moderate-intensity walking was sufficient to reduce the metabolic risk profile, and reduced blood pressure in postmenopausal women [26].

In this study, 60 min after the walk on day 7, salivary cortisol was reduced by 1.87 μg/dL (*p* = 0.455) in the park exposure group, and DBP was reduced by 6 mm Hg (*p* = 0.002), as well as showing a positive effect of the physical activity on DBP. Walking in both city environments and performing all measurements took place at the same time of day, and therefore diurnal variability of cortisol should not have had any significant effect on the obtained results.

## 5. Conclusions

This controlled field study extends the evidence for a biological pathway of the beneficial effects of green space exposure on health, which support the notion of stress relief and restoration as possible mechanisms through which natural environments confer health benefit. Our findings indicate that physical activities in the park environment, coherent with stress reduction, can have a better restorative effect on post-MI patients’ cardiac function, compared to the urban street environment with higher noise and air pollution levels. However, further research with a bigger sample size and a longer treatment is required to confirm our conclusion. Understanding of the mechanisms could expand the appropriate use of the green environment to decrease stress and improve cardiac function, thereby increasing the effectiveness of contact with nature at a population level for the prevention of CVD.

## Figures and Tables

**Table 1:** The characteristics of the study subjects at baseline and after exposure to urban street and park environment.

Baseline Characteristics	Urban Street Mean (SE)	Park Environment Mean (SE)	*p* *
Men, n (%)	6 (60)	7 (70)	0.500
Age, years	66.0 (12.5)	58.5 (12.2)	0.162
Body mass index	27.9 (1.8)	27.9 (4.9)	0.264
CAD anamnesis, years	9.3 (8.8)	8.8 (11.7)	0.353
Time after the last hospitalization for CAD, years	1.16 (0.6)	0.90 (0.4)	0.176
Systolic BP, mm Hg	134.7 (6.8)	135.9 (5.5)	0.382
Diastolic BP, mm Hg	80.3 (3.3)	81.4 (1.7)	0.398
Heart rate, bpm	77.7 (4.0)	71.3 (3.8)	0.125
Cortisol, μg/dL **^a^**	12.91 (2.1)	13.3 (1.6)	0.375 **
Positive effects, score	26.0 (1.1)	26.2 (1.8)	0.463 **
Negative effects, score	15.9 (1.9)	13.1 (0.8)	0.198
Air temperature during walking, °C	18.0 (0.53)	14.11 (1.66)	0.092
Relative humidity during walking, %	75.0 (1.23)	83.4 (2.85)	0.005
NO_2_ during walking, µg/m^3^	24.15 (1.69)	20.31 (0.93)	0.026
PM_2.5_ during walking, µg/m^3^	24.64 (0.97)	18.23 (0.85)	0.001
Noise during walking, dBA	65.20 (1.31)	46.17 (0.78)	0.000

**^a^** *t* test used for logarithmic data; * exact one-tailed *p*-value of the Mann-Whitney U test; ** one-tailed *p*-value of the *t*-test.

**Table 2:** The relationships between cortisol, mood, and hemodynamic parameters (Spearman’s correlation coefficient).

Measurements	Cortisol 1st Day	Cortisol Slope	Cortisol 1–7 Day Changes	PE	NE
Cortisol baseline	1				
Cortisol slope	−0.252	1			
Cortisol 1–7 day changes	−0.018	−0.277	1		
Positive effects	−0.270	−0.037	0.038	1	
Negative effects	0.471 *	0.110	−0.239	−0.179	1
SBP, mm Hg	−0.363 **^†^**	0.285	−0.131	0.483 *	−0.160
DBP, mm Hg	−0.090	0.180	−0.138	0.151	−0.168
HR, bpm	0.061	−0.375 **^†^**	−0.132	−0.034	−0.002
SBP 1–7 day changes	−0.069	−0.296	−0.056	−0.402 *	0.258
DBP 1–7 day changes	0.142	−0.514 *	−0.166	0.130	0.095
HR 1–7 day changes	0.189	−0.047	−0.158	0.082	0.385 **^†^**

**^†^** one-side *p* < 0.1; * one-side *p* < 0.05.

**Table 3:** The difference of hemodynamic parameters, cortisol, and mood after the first day walk in urban and park environments.

Measurements	Difference 1 min after Walk		Difference 60 min after Walk	
	Median	*p* ^†^ Value	Median	*p* ^†^ Value
**Urban exposure**				
SBP, mm Hg	16.75	0.008	−3.00	0.473
DBP, mm Hg	10.00	0.008	5.50	0.156
HR, bpm	18.75	0.004	4.75	0.371
Cortisol (μg/dL)	−0.84	0.358	−1.66	0.188
Positive effects	0	0.368 **^††^**	−0.50	0.405 **^††^**
Negative effects	−0.5	0.063	0	0.500
**Green exposure**				
SBP, mm Hg	13.50	0.023	1.00	0.186
DBP, mm Hg	0.50	0.227	1.50	0.318
HR, bpm	19.00	0.010	2.00	0.238
Cortisol (μg/dL)	−1.93	0.042	−2.89	0.037
Positive effects	−3.00	0.002 **^††^**	−2.00	0.012 **^††^**
Negative effects	−0.5	0.133	−1.00	0.031

**^†^** exact one-tailed *p*-value of the Mann-Whitney U test; **^††^** one-tailed *p*-value of the *t*-test.

**Table 4:** Changes in hemodynamic parameters, cortisol, and mood between the first and seventh days exposure in different environments.

Changes between Day 1 and Day 7	Urban Changes Median	*p* ^†^ Value	Park Changes Median	*p* ^†^ Value
**Baseline**				
SBP, mm Hg	8.00	0.336	−0.50	0.456
DBP, mm Hg	0	0.453	−4.00	0.031
HR, bpm	−2.00	0.348	1.50	0.500
Cortisol level	0.59	0.216	0.96	0.161
Positive effects	−1.00	0.414	0.5	0.422
Negative effects	−2.00	0.002	0	0.297
**60 min after the walk**				
SBP, mm Hg	2.00	0.156	−3.00	0.131
DBP, mm Hg	2.00	0.336	−6.00	0.002
HR, bpm	−16.00	0.109	0	0.305
Cortisol level	0.55	0.432	−1.87	0.455
Positive effects	−1.50	0.387	0.50	0.322
Negative effects	−1.50	0.008	0	0.453

**^†^** exact one-tailed *p*-value of the Wilcoxon test.
